# Benefits of a laparoscopic approach for second colorectal resection after colectomy or proctectomy –a retrospective study-

**DOI:** 10.1186/s12893-023-02111-6

**Published:** 2023-08-04

**Authors:** Hiroaki Nozawa, Kazuto Sasaki, Shigenobu Emoto, Koji Murono, Yuichiro Yokoyama, Hirofumi Sonoda, Yuzo Nagai, Shinya Abe, Soichiro Ishihara

**Affiliations:** Department of Surgical Oncology, The University of Tokyo, 7-3-1 Hongo, Bunkyo-ku, Tokyo, 113-8655 Japan

**Keywords:** Laparoscopic surgery, Surgical outcomes, Second colorectal surgery, Blood loss, Bowel function recovery

## Abstract

**Background:**

A laparoscopic approach generally provides several benefits in patients who undergo colon or rectal surgery without jeopardizing oncological outcomes. However, there is a paucity of studies on comparative outcomes of laparoscopic versus open approaches for second primary colorectal lesions after colectomy or proctectomy.

**Methods:**

From patients with colorectal disease who underwent surgery between 2008 and 2022 at our hospital, we collected 69 consecutive patients who had previous colorectal surgery for this retrospective study. Based on the second surgery approach (laparoscopic or open), patients were classified into the Lap (*n* = 37) or Op group (*n* = 32). Patients’ baseline data and perioperative and postoperative outcomes were compared between the two groups.

**Results:**

Four patients (11%) of the Lap group needed conversion to laparotomy. The intraoperative blood loss was lower in the Lap group than the Op group (median: 45 ml vs. 205 ml, *p* = 0.001). The time to first bowel movement was shorter in the Lap group than the Op group (median: 2.8 days vs. 3.6 days, *p* = 0.007). The operative time, frequencies of postoperative morbidities, and overall survival did not differ between the two groups.

**Conclusion:**

Laparoscopic surgery appeared feasible and beneficial for selected patients undergoing second colorectal resection after colectomy or proctectomy regarding blood loss and bowel function recovery without affecting other outcomes.

## Background

Remarkable progress in laparoscopic surgery has been seen in a variety of field including for the colorectum, with fewer intraoperative bleeding, fewer complications, faster postoperative recovery, better cosmesis, and satisfactory oncological outcomes [1–10], except for intriguing findings regarding resection margin among pivotal randomized control trials (RCTs) that compared laparoscopic and open surgeries for rectal cancer [6–9]. Encouraged by the results of these RCTs, surgeons have applied minimally invasive techniques to more complicated procedures. For example, total proctocolectomy plus ileal pouch-anal anastomosis, standard surgical procedures for familial adenomatous polyposis (FAP) and ulcerative colitis (UC), can be done via a laparoscopic approach with similar advantages over the open method, as demonstrated by several cohort studies and a few RCTs [11–16]. Advanced colorectal cancer (CRC) adhering to or invading adjacent organs is another complex surgical target that can be resected laparoscopically [17, 18]. The feasibility of minimally invasive pelvic lymphadenectomy in advanced lower rectal cancer was also demonstrated by previous reports [19, 20].

CRCs can arise in a multi-centric and/or metachronous manner [21]. Even after surgical resection of CRC, another primary CRC can develop with incidence ratios of 2–9% [21, 22]. Apart from genetically predisposed patients, an elevated risk for metachronous CRC is observed in patients with factors such as synchronous multiple CRCs, CRC with high microsatellite instability, and environmental factors [22–25]. Laparoscopic approach in second colorectal surgery in such patients is considered more challenging than that in surgery- naïve patients because of potential intra-abdominal adhesion, resulting in an elevated risk of unexpected trauma [26]. Unfortunately, there is a paucity of studies that compare laparoscopic and open surgeries in this setting. Therefore, we compared the surgical outcomes of laparoscopic vs. open resection of the colorectum after colectomy or proctectomy in the current study.

## Methods

### Patients

Consecutive patients were reviewed retrospectively who underwent surgical resection (designated as ‘second’ surgery) of primary colorectal lesions with a past history of colectomy or proctectomy (designated as ‘first’ surgery) at the University of Tokyo Hospital between 2008 and 2022. Here, reoperation aimed at managing complications of preceding colorectal surgery, e.g. anastomotic leakage or stenosis, or bowel obstruction, local resection of tumors near the anus, and surgical cases of FAP or UC were excluded. Recurrent tumors involving the colorectum were also excluded. Patients who underwent emergency surgery, total pelvic exenteration, had a history of two or more colorectal resections, or simultaneous resections of other sites were also excluded. According to the second surgery approach, patients were then subdivided into the laparoscopic (“Lap”) or open (“Op”) group. Here, the former included cases of conversion to laparotomy.

This study was approved by the ethics committee of the University of Tokyo Hospital (reference: 3252-15). We obtained written informed consent from all patients, and provided them the opportunity to opt-out for inclusion in this study.

### Surgery

Colonoscopy, contrast-enhanced computed tomography (CT) scans, and CT colonoscopy are routinely performed before elective surgery at our department. To visualize the vascular anatomy, three-dimensional angiography was also performed, which help us to plan the ligation of mesenteric vessels [27].

Several teams of board-certified surgeons were involved in laparoscopic or open resection of primary colorectal lesions in our hospital. After the standardization of laparoscopic surgery for CRC (around 2012), laparoscopic colorectal surgery in complicated situations, such as simultaneous resection with other organs or previous bowel resections, were gradually applied. Detailed open or laparoscopic procedures of colorectal resection were described previously [28]. From 2019, indocyanine green (ICG) fluorescence angiography was used to visualize tissue perfusion before anastomosis in selected patients.

### Data collection

Clinical data and demographics were collected on sex, age, body mass index, preoperative levels of serum albumin, and hemoglobin, comorbid illness, chronic anti-thrombotic therapy, and preoperative chemotherapy for colorectal malignancy from medical records. In addition to the type and location of disease and surgical approaches in the first and second colorectal surgeries, second surgery-related parameters, namely, period (2008–2014 or 2015–2022), surgical procedure, resection including previous anastomosis, operative time, conversion to open laparotomy, intraoperative ICG fluorescence imaging, estimated volume of blood loss during surgery, first bowel movement after surgery, and postoperative morbidities were collected. To evaluate postoperative complications, the Clavien-Dindo classification was used [29]. Resected specimens were evaluated for bowel length, and in case of malignant disease, resection margin, tumor stage according to the American Joint Committee on Cancer and International Union Against Cancer tumor-node-metastasis grading system [30], and the number of lymph nodes retrieved were recorded. Overall survival (OS) was defined as the time from the second colorectal surgery to death from any causes.

### Statistics

Continuous data were compared by the unpaired t or Mann-Whitney U test, whereas categorized variables were compared by the Fisher’s exact or chi-squared test with or without Yates’ correction. We used Kaplan-Meier methods to draw estimated survival curves. The difference in OS between patient groups was examined using the log rank test. JMP software ver.16.2.0 (SAS Institute Inc., Cary, NC, USA) was used to perform all analyses. All reported *p* values were two-sided, and considered significant if less than 0.05.

## Results

### Patient overview

**Fig. 1:**
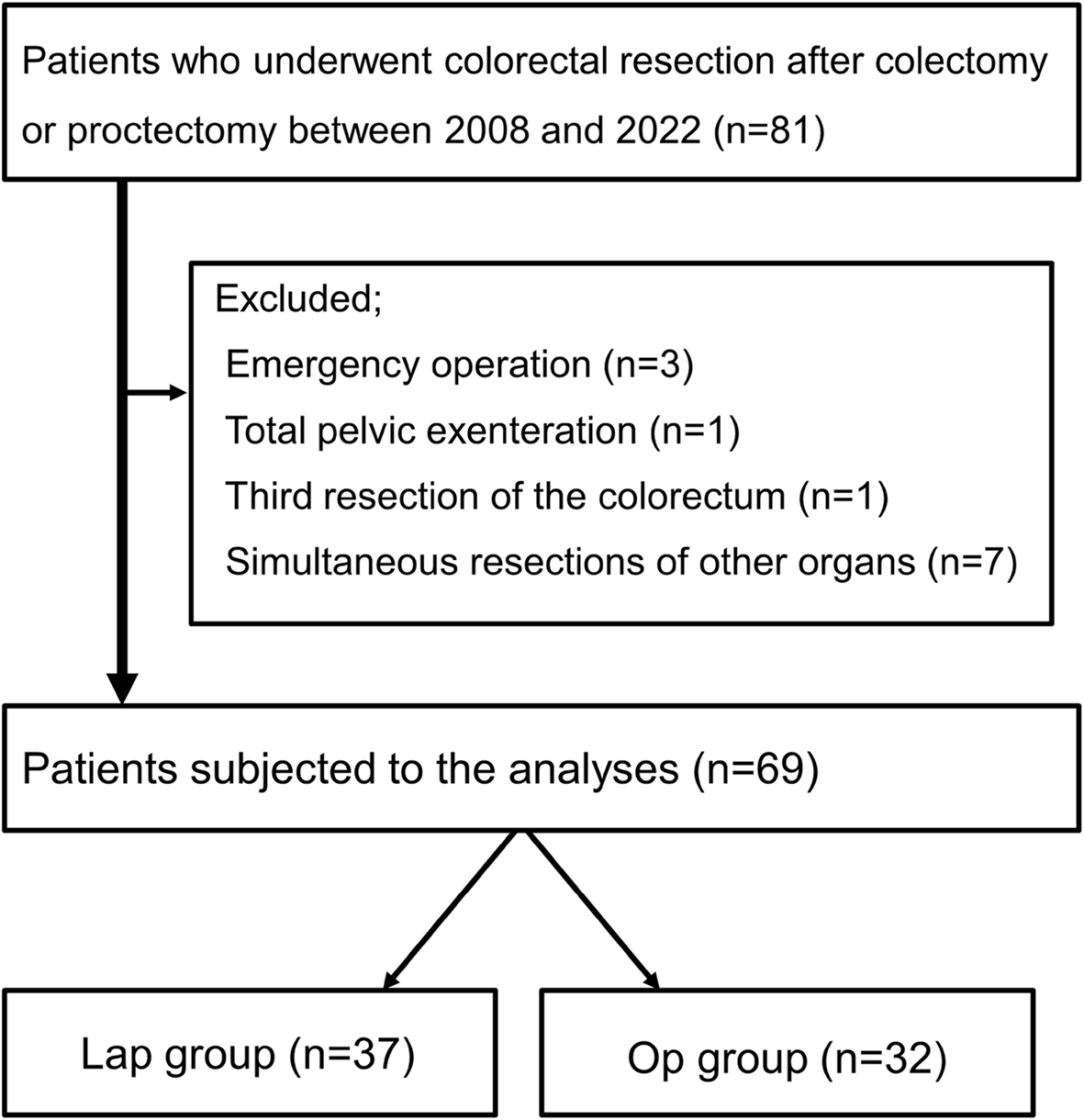
Flow chart of selecting study subjects

During the study period, a total of 81 patients underwent surgical resection for second primary lesions of the colorectum after colectomy or proctectomy. As shown in Fig. 1 and 69 patients were analyzed after excluding four patients who did not fulfill the study criteria.

The preoperative clinical variables are summarized in Table 1. The laparoscopic approach was predominantly selected in the second colorectal resection in the late period (2015–2022). Moreover, more patients in the Lap group were on chronic antithrombotic medication than those in the Op group (35% vs. 6%, *p* = 0.009). No difference was noted in other background characteristics between the groups.

**Table 1:** Baseline characteristics of patients according to second surgical approach

		Lap	Op	
Variable		(*n* = 37)	(*n* = 32)	*p* value
Period	2008–2014	6 (16%)	24 (75%)	< 0.0001
	2015–2022	31 (84%)	8 (25%)	
Sex	Male	24 (65%)	23 (72%)	0.53
Age, year	Mean ± SD	71.0 ± 11.5	70.2 ± 9.7	0.76
BMI, kg/m^2^	Mean ± SD	24.6 ± 3.5	23.0 ± 3.4	0.064
Hemoglobin, g/dL	Mean ± SD	12.0 ± 1.9	12.0 ± 1.9	1.00
Albumin, g/dL	Mean ± SD	3.7 ± 0.5	3.7 ± 0.5	0.78
Comorbidity	Diabetes	8 (21%)	10 (31%)	0.53
	COPD	0 (0%)	3 (9%)	0.095
	Hypertension	19 (51%)	13 (41%)	0.37
	Ischemic heart disease	4 (11%)	3 (9%)	1.00
	CKD	2 (5%)	2 (6%)	1.00
	Cerebrovascular disease	5 (14%)	1 (3%)	0.21
	CNS disorder	1 (3%)	3 (9%)	0.33
	Chronic hepatitis / cirrhosis	0 (0%)	1 (3%)	0.46
	Autoimmune disease	0 (0%)	1 (3%)	0.46
Anti-thrombotic therapy		13 (35%)	2 (6%)	0.009
Preoperative systemic chemotherapy		0 (0%)	1 (3%)	0.46

*SD* Standard deviation, *BMI* Body mass index, *COPD* Chronic obstructive pulmonary disease, *CKD* Chronic kidney disease, *CNS* Central nervous system

Profiles of the first and second surgical diseases were compared between the Lap and Op groups (Table 2). The majority of patients received first or second colorectal surgery for malignancy. The type and location of disease in the first operation were similar between the two groups. Most patients (94%) received open surgery for the first colorectal disease in the Op cohort; in contrast, the laparoscopic approach was previously selected in 43% of the Lap cohort (*p* = 0.0007). No marked differences were observed in the type of disease for the second surgery between the two groups. The location of the second colorectal disease was also similarly distributed.

**Table 2:** Details of colorectal diseases treated by the first and second surgeries according to second surgical approach

		Lap	Op	
Variable		(*n* = 37)	(*n* = 32)	*p* value
a) First operation				
Type of disease				1.00
Benign	Perforation due to bowel obstruction	0 (0%)	1 (3%)	
	Iatrogenic perforation	0 (0%)	1 (3%)	
Malignant	Carcinoma	36 (94%)	29 (91%)	
	Neuroendocrine neoplasm	1 (3%)	0 (0%)	
Unknown		0 (0%)	1 (3%)	
Location^a^	Cecum/appendix	2 (5%)	1 (3%)	0.90
	Ascending colon	7 (18%)	4 (12%)	
	Transverse colon	4 (11%)	4 (12%)	
	Descending colon	0 (0%)	2 (6%)	
	Sigmoid colon	10 (26%)	13 (38%)	
	Rectum	15 (39%)	10 (29%)	
Approach	Open	21 (57%)	30 (94%)	0.0007
	Laparoscopic	16 (43%)	2 (6%)	
b) Second operation				
Type of disease				0.94
Benign	Diverticulum	1 (3%)	0 (0%)	
	Benign neoplasm	0 (0%)	1 (3%)	
Malignant	Carcinoma	35 (94%)	30 (94%)	
	Neuroendocrine neoplasm	1 (3%)	1 (3%)	
Location^a^	Cecum/appendix	1 (3%)	2 (6%)	0.81
	Ascending colon	6 (16%)	11 (33%)	
	Transverse colon	15 (41%)	9 (27%)	
	Descending colon	3 (8%)	1 (3%)	
	Sigmoid colon	8 (22%)	6 (18%)	
	Rectum	6 (16%)	4 (12%)	

^a^Multiple disease locations were found in several patients

### Perioperative outcomes in the second colorectal surgery

Surgical procedures and findings at the second operation are presented in Table 3. The median interval between the two operations was more than 10 years in both groups. There was no significant difference in the type of surgery between the two groups. The median operative time in the Lap cohort was 24 min longer than that in the Op cohort; the difference was not significant. Four cases underwent conversion (11% of the Lap group); all received open surgery for the first colorectal lesion. Among them, three patients required conversion to laparotomy for a limited view due to dense adhesions in the peritoneum, and in the other patient with transverse colon cancer, conversion to the laparotomy procedure was necessary in order to control major bleeding from the greater omentum behind the adhesions. Intraoperative ICG fluorescence imaging was used in only 16% of the Lap group. The Lap cohort had significantly less blood loss than the Op cohort (median: 45 mL vs. 205 mL, *p* = 0.001).

**Table 3:** Perioperative parameters according to second surgical approach

		Lap	Op	
Variable		(*n* = 37)	(*n* = 32)	*p* value
Interval from first operation, month	Mean ± SD	122 ± 101	139 ± 113	0.51
Colon / rectal surgical procedure	Right-sided colectomy	15 (41%)	17 (53%)	0.84
	Transverse colectomy	6 (16%)	4 (13%)	
	Left-sided colectomy	3 (8%)	1 (3%)	
	Sigmoid colectomy	0 (0%)	2 (6%)	
	Anterior resection / intersphincteric resection	11 (30%)	5 (16%)	
	Abdominoperineal resection	2 (5%)	3 (9%)	
Resection including previous anastomosis	Yes	13 (35%)	12 (38%)	0.84
Operative time, min	Median (IQR)	271 (227–391)	247 (192–349)	0.28
Conversion		4 (11%)	-	N/E
Intraoperative use of ICG		6 (16%)	0 (0%)	0.027
Blood loss, mL	Median (IQR)	45 (15–195)	205 (121–418)	0.001
Time until first bowel movement, day	Mean ± SD	2.8 ± 1.2	3.6 ± 1.2	0.007
Complications, CD grade 2-^a^	Any	10 (27%)	12 (38%)	0.35
	Leakage	0 (0%)	1 (3%)	0.46
	Bleeding	2 (5%)	0 (0%)	0.21
	Small bowel obstruction	3 (8%)	6 (19%)	0.29
	SSI, incisional	0 (0%)	2 (6%)	< 0.0001
	SSI, organ/peritoneal	2 (5%)	4 (13%)	0.41
	Cholecystitis	1 (3%)	0 (0%)	1.00
	Urinary tract infection	3 (8%)	0 (0%)	0.24
	CRBSI	1 (3%)	1 (3%)	1.00
	Pneumonia	1 (3%)	1 (3%)	1.00
	Acute kidney injury	1 (3%)	0 (0%)	1.00

^a^Multiple complications were observed in several patients

*N/E* Not evaluated, *SD* Standard deviation, *IQR* Interquartile range, *ICG* Indocyanine green, *CD* Clavien-Dindo classification, *SSI* Surgical site infection, *CRBSI* Catheter-related blood stream infection

Postoperative parameters were compared between the Lap and Op groups (Table 3). The median time to first bowel movement was shorter in the Lap cohort than the Op cohort (2.8 days vs. 3.6 days, *p* = 0.007). There was no significant difference in individual or overall morbidities between the groups except for incisional surgical site infection observed only in the Op cohort. Anastomotic leakage was noted in a female patient in the Op group, which was conservatively treated; she was discharged with full activities of daily living. On the other hand, postoperative bleeding of Clavien-Dindo classification grade 2 was observed only in the Lap group (6%), which may be related to the high frequency of chronic anti-thrombotic therapy. There was no death within one month of surgery in either group.

Regarding pathological findings, lengths of resected specimen did not differ between the Lap and Op groups. In cases of colorectal malignancies, the resection margin was free from tumors in all patients. Laparoscopic surgery resulted in a higher lymph node yield than open surgery (median: 21 vs. 14, *p* = 0.13). We found no significant variation in tumor stages between the two groups, but distant metastasis was only present in the Op group (Table 4).

**Table 4:** Oncological parameters according to second surgical approach

		Lap	Op	
Variable		(*n* = 37)	(*n* = 32)	*p* value
Length of bowel resection, mm	Mean ± SD	270 ± 96	245 ± 130	0.37
Resection margin^a,b^	Positive	0 (0%)	0 (0%)	1.00
Number of harvested lymph nodes^a^	Median (IQR)	21 (9–33)	14 (5–26)	0.13
Pathological T^a,c^	-T1	17 (47%)	10 (32%)	0.41
	T2	1 (3%)	1 (3%)	
	T3	14 (39%)	11 (36%)	
	T4	4 (11%)	9 (29%)	
Pathological N^a^	N0	28 (78%)	23 (74%)	0.94
	N1	6 (17%)	6 (19%)	
	N2	2 (5%)	2 (7%)	
Distant metastasis^a^	Present	0 (0%)	3 (9%) ^d^	0.095
Stage^a,c^	0-I	16 (45%)	10 (32%)	0.56
	II	12 (33%)	12 (39%)	
	III	8 (22%)	6 (20%)	
	IV	0 (0%)	3 (9%)	

^a^calculated for patients with colorectal malignancy

^b^evaluated for colorectal lesions

^c^more advanced stage was counted in patients with multiple tumors

^d^liver metastasis in three, peritoneal metastasis in two, and lung metastasis in one

*SD* Standard deviation, *IQR* Interquartile range

### Long-term outcomes after the second colorectal surgery

**Fig. 2:**
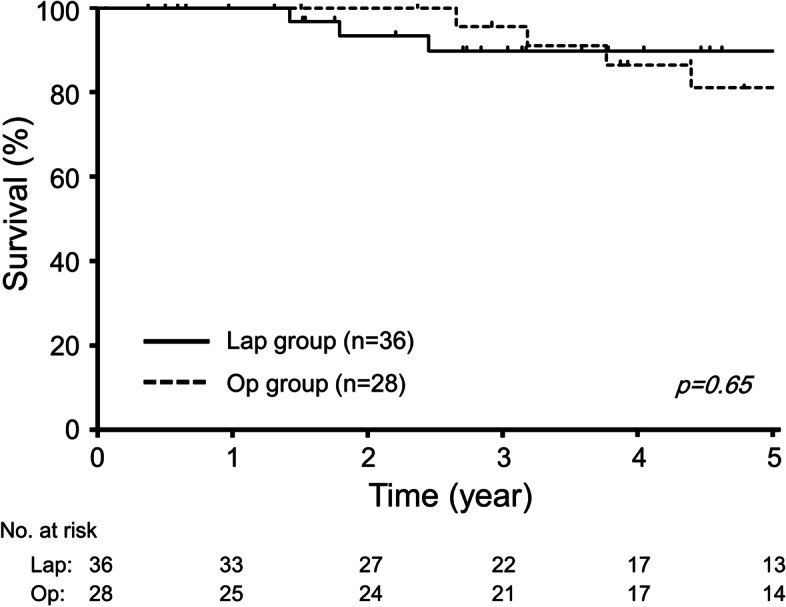
The Kaplan-Meier curves of overall survival of 64 patients according to the second surgical approach for localized colorectal tumor

Survival outcomes were analyzed after excluding two patients with benign disease and three patients with distant organ metastasis at the second colorectal surgery. The median follow-up for this study population (64 patients) was 52.6 months. There were eight deaths (13%) in the follow-up period. The cause of death was related to second colorectal disease in two patients in the Lap cohort, and three patients in the Op cohort. OS rates at three years and five years for the entire cohort were 92% and 85%, respectively. As shown in Fig. 2, OS in the Lap cohort did not differ from that in the Op cohort (*p* = 0.65).

## Discussion

This is the first study to compare laparoscopic and open approaches for second colorectal surgery in patients who had undergone previous colectomy or proctectomy. We demonstrated that the laparoscopic approach markedly reduced blood loss during operation and facilitated return of bowel function without affecting other clinicopathological parameters in this setting. Similar to the current study, we recently reported the comparative outcomes of laparoscopic and open ileal-anal pouch surgery after total colectomy in FAP or UC patients who underwent staged operations; the laparoscopic approach for the pouch surgery was shown to reduce the volume of blood loss and time to first bowel transit without significant disadvantages [31].

In resections of multiple colorectal segments, it is crucial to preserve blood supply to the remaining bowel [32]. Based on preoperative CT findings and three-dimensional angiography as described [27], the levels of main vessel ligation and lymphadenectomy were planned to avoid unexpected bowel ischemia or anastomotic leakage. Indeed, we encountered only one case of leakage out of 69 patients (1.4%), a rate lower than those in a recent RCT and a national database report in Japan (3.6–10.2%) [33, 34]. As performed in several patients in our study, the application of ICG-guided surgery may be useful in laparoscopic surgery for metachronous CRC [35].

The open conversion rate in the Lap cohort was 11% in the current study. It seems satisfactory as it is comparable to the reported conversion rate range (5–29%) in previous RCTs for laparoscopic surgery for primary rectal or colon cancer [2–9]. Notably, there were no conversion cases when considering patients who had undergone preceding colorectal surgery via a laparoscopic approach presumably owing to less intraabdominal adhesion [36–38]. The number of patients receiving minimally invasive surgery is increasing worldwide [39, 40]. This trend will lead to more opportunities for repeated laparoscopic surgery for colorectal lesions.

In the present study, more lymph nodes were retrieved in the Lap group than in the Op group. However, pivotal RCTs on coloncancer reported no significant differences in lymph node yields between the two surgical approaches [2–5]. Therefore, the present results may be attributed to a possible bias in that patients with fewer intraabdominal adhesions were selected for a second laparoscopic surgery.

The findings of the current study were limited due to its retrospective nature and potential biases. This was a single institute series, and the total number of patients analyzed was relatively small, which might result in type 2 errors in several outcomes. In addition, the indication for laparoscopic surgery may have been biased based on findings of preoperative image studies and previous surgery records. Several patient characteristics were not balanced, such as period of surgery and the preceding colorectal surgery approach between the two patient groups. Furthermore, a higher frequency of anti-thrombotic therapy was noted in the Lap cohort, but blood loss was lower than in the Op cohort; therefore, this baseline disparity does not essentially negate our main findings. Lastly, there were only a few patients who received preoperative therapy, and its additional impact on surgical outcomes could not be fully addressed.

## Conclusions

It was demonstrated that compared to open surgery, a laparoscopic approach for a second surgery of the colorectum after colectomy or proctectomy is safe and beneficial for selected patients because of a reduction of intraoperative blood loss and faster return of bowel function, even under suboptimal conditions. Our findings need to be verified using a larger number of patients in multiple institutes.

## Data Availability

The datasets generated during and/or analyzed during the current study are not publicly available because they are derived from the patient database of the hospital and hence subject to confidentiality, but are available from the corresponding author on reasonable request.

## Abbreviations

CRC: Colorectal cancer
CT: Computed tomography
FAP: Familial adenomatous polyposis
ICG: Indocyanine green
OS: Overall survival. RCT:randomized control trial
UC: Ulcerative colitis
